# Asthma daytime and nighttime symptom diaries: content validity and qualitative exploration of meaningful change in patients with moderate-to-severe asthma

**DOI:** 10.1186/s41687-026-01079-0

**Published:** 2026-05-28

**Authors:** Tom Keeley, Katie Forde, Hannah Elwick, Adam Gater, Sophie Lawrie, Agkreta Leventi, Rafael Alfonso-Cristancho

**Affiliations:** 1 Digital Measures, RWDMA, Development Sciences, GSK, London, UK; 2 Patient-Centered Outcomes, Adelphi Values, Bollington, Cheshire, UK; 3 Global Real-World Evidence and Health Outcomes Research, GSK, Collegeville, PA USA

**Keywords:** Patient-reported outcome, Moderate asthma, Severe asthma, Content validity, Meaningful change, Asthma Daytime Symptom Diary, Asthma Nighttime Symptom Diary

## Abstract

**Background:**

Asthma symptom severity can be assessed using the Asthma Daytime and Nighttime Symptom Diaries (ADSD and ANSD), which provide an accurate and standardized patient-reported option for symptom assessment during different periods of the day (i.e. daytime and nighttime), as these can vary significantly. Earlier development and validation work for the ADSD and ANSD was conducted in a broad (i.e. ranging from mild to severe) asthma population, and a US Food and Drug Administration qualification statement noted the need for further evaluation of their measurement properties, including content validity. The current study, which builds upon earlier work, was designed to ensure that the ADSD and ANSD are content-valid in patients with moderate-to-severe asthma.

**Methodology:**

Qualitative semi-structured concept elicitation and cognitive debriefing interviews were conducted in adults with moderate-to-severe asthma to investigate understanding of ADSD and ANSD instructions, items, recall period, response options, and concept relevance. Changes in total and item scores that were meaningful to patients were explored. General feedback on the measures was also requested.

**Results:**

The study recruited 15 patients with moderate-to-severe asthma. Content validity findings were consistent with previous studies, supporting inclusion of six core symptoms (i.e. wheezing, cough, difficulty breathing, shortness of breath, chest tightness and chest pain) as items in the ADSD and ANSD that occur during a typical day or night and impact upon patient well-being and functioning. A high level of understanding and relevance of the diary items to patients was demonstrated; instructions were confirmed to be easy to follow. In supportive analyses, meaningful levels of improvement on the ADSD and ANSD ranged from 0.5 to 3.5 and 0.2 to 5.0 respectively. Meanwhile, meaningful levels of worsening on the ADSD and ANSD ranged from 1.0 to 5.8 and 0.5 to 6.7 respectively.

**Conclusions:**

The ADSD and ANSD are content-valid measures for the measurement of daytime and nighttime symptoms and their severity in patients with moderate-to-severe asthma. Additional confirmatory studies are required to confirm the psychometric properties of the ADSD and ANSD in a moderate-to-severe population and to quantitatively estimate the threshold for meaningful improvement and worsening.

**Supplementary Information:**

The online version contains supplementary material available at 10.1186/s41687-026-01079-0.

## Background

Asthma symptom severity may range from mild (i.e. well-controlled with low-intensity treatment; Global Initiative for Asthma [GINA] steps 1 or 2) to moderate (i.e. well-controlled with higher-intensity treatment; GINA steps 3 or 4) or severe (remaining uncontrolled despite optimised high-dose treatment; often categorized as GINA step 5) [1]. Patients with poor symptom control often have reduced quality of life, with negative impacts on social, physical, and emotional function, work productivity, and healthcare resource use [2].

Many asthma symptoms, such as shortness of breath or chest pain, can best be assessed directly by patients themselves using patient-reported outcome (PRO) measures [3] such as the Asthma Control Questionnaire and St George’s Respiratory Questionnaire [4–6]. Changes in symptom scores can be used to identify meaningful score difference, which is often determined based on a patient’s assessment of what constitutes clinically meaningful change (improvement or deterioration) for them personally (i.e., meaningful within-patient change) [7]. Asthma PRO measures can provide valuable insights into the subjective impact of symptoms on perceived quality of life and are also useful for clinical decision-making [4, 8]. Currently available PRO measures, which have been validated for use in asthma clinical trials and practice settings, focus more on asthma control and quality of life as a whole rather than symptoms and their severity. The PRO measures currently available do not independently assess night symptoms, which are a key issue for many patients [4–6, 8], with symptoms reportedly worsening in the early hours of the morning (i.e., during the nighttime period) [9]. To fully appreciate the individual experience of asthma, there is a need to capture the nuances of both daily and nightly symptom variation and understand patterns of treatment response over time [8].

The Asthma Daytime Symptom Diary (ADSD) and Asthma Nighttime Symptom Diary (ANSD) were designed to address the gap in standardized PRO measures of asthma symptoms and their severity [5, 10]. Each diary consists of six items evaluating the severity of a set of core asthma symptoms, including difficulty breathing, wheezing, shortness of breath, chest tightness, chest pain, and cough. Patients are required to rate each item on an 11-point numeric rating scale, ranging from 0 (“None”; i.e., a low score) to 10 (“As bad as you can imagine”; i.e., a high score). The ADSD is designed to be completed daily, before going to bed, and asks respondents to rate the severity of their asthma symptoms during that particular day, while the ANSD is completed daily upon waking and asks respondents to rate the severity of symptoms during the previous night [11]. Data were captured in qualitative (*n* = 120) [10] and quantitative (*n* = 219) [11] studies including United States (US)-based patients with mild-to-severe persistent asthma (i.e. in a broad asthma population) aged 12 years and above. These data were subsequently utilized to inform the initial development, establish content validity, and evaluate the cross-sectional measurement properties of the ADSD and ANSD. The measures were developed in line with the current recommendations at the time for PRO measures for asthma from published literature and US Food and Drug Administration (FDA) guidelines, to ensure reliable, valid and accurate outcome measurement [11].

Use of the ADSD and ANSD in drug development and regulatory review was supported in the FDA qualification statement, but the need for further evaluation of their measurement properties, specifically ability to detect change overtime and ability to interpret meaningful within-patient change, was noted. A clinical outcome assessment is only considered fit for purpose when the level of validation is sufficient to support its specific context of use [12]. Given the wide range of asthma severity types (i.e. from mild, to moderate, to severe), it is necessary to establish the validity of clinical outcome assessments such as the ADSD and ANSD specifically within the moderate-severe asthma population, as this will likely differ to that of a broad asthma population.

Quantitative evaluation of the ADSD and ANSD measurement properties has been performed in a real-world observational study and a randomized controlled trial of patients with moderate-to-severe asthma [13]. This article summarizes a detailed qualitative assessment of the ADSD and ANSD, providing confirmatory evidence of population and context of use-specific content-validity in patients with moderate-to-severe asthma [14], and also qualitative evidence to contribute to the estimation of thresholds for clinically meaningful within-patient changes in ADSD and ANSD scores (score range: 0–10) in this population [15].

## Methods

**Fig. 1:**
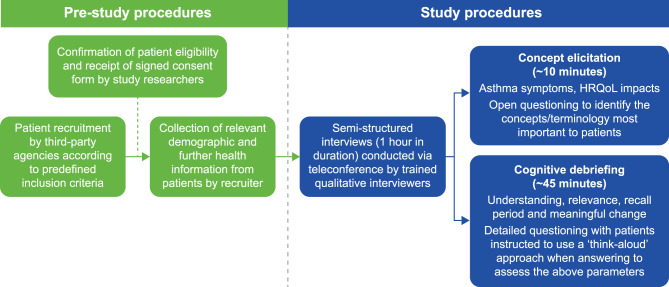
Brief overview of pre-study and study procedures. HRQoL, health-related quality of life

In this study, qualitative semi-structured interviews were conducted in a population of adult patients with a verified diagnosis of moderate-to-severe asthma (GSK ID: 214566). The three main objectives were to assess the following aspects relating to the ADSD and ANSD: 1) content validity and key relevant concepts for this patient population; 2) patient understanding and interpretation; and 3) qualitative exploration of meaningful change. Details of the development process for the ADSD have previously been published [10], and information relating to the ADSD and ANSD can be found at the Critical Path Institute’s Patient-Reported Outcome Consortium site (https://www.c-pathcoas.org/adsd-ansd). An overview of pre-study and study procedures is shown in Fig. 1.

### Study sample

Patients were recruited into the study by two third-party recruitment agencies, via pre-existing patient panels/databases in the US, according to the prespecified inclusion criteria. Patients were eligible if they were aged ≥ 16 years at the time of enrollment; diagnosed with asthma ≥ 2 years previously (self-reported); receiving GINA step 4–5 medications (defined as use in the past 3 months of a medium- or high-dose inhaled corticosteroid and a long-acting β_2_-agonist, and add-on controller therapy [inhaled long-acting muscarinic antagonist and/or oral leukotriene receptor antagonist and/or oral corticosteroid and/or biologic therapy for asthma]); and had experienced ≥ 1 severe exacerbation of asthma in the previous year (self-reported; defined as the requirement for addition or increase in the dose of oral corticosteroid treatment, and/or injection of corticosteroid, and/or visits to the emergency room and/or hospitalization due to asthma symptoms). Patients with a diagnosis of a respiratory condition other than asthma (except for allergies or rhinitis), any significant lung, heart, gastrointestinal or neurological disease, or who had COVID-19 in the past 3 months (self-reported) were excluded.

It is recommended that sample sizes are determined based on the concept of ‘conceptual saturation’ in qualitative research aiming to provide a comprehensive understanding of a research topic. Saturation is commonly defined as the point at which no new concept-relevant information is identified (i.e., that all domains of importance to a patient relevant to the higher-level concept have been elicited) with the repeated collection of data. In a homogenous population, conceptual saturation may be achieved in 10–12 interviews [16, 17]; sample sizes of this magnitude have also previously been assessed as sufficiently reliable to generating a range of patient responses and experiences and to explore respondent understanding and comprehension [18].

### Study procedures

The study sampling approach was purposive, as specific criteria were applied and recruitment quotas employed. Patients completed two informed consent forms; one consenting to have their health information (i.e., information collected on the patient-completed screener form and patients’ evidence of their asthma medication) shared with the study team to confirm their eligibility, and one for eligible patients, consenting to participate in the interview. Once the study team confirmed patient eligibility and obtained signed informed consent for interview participation, the recruiter collected relevant demographic and further health information from patients over the phone, using a demographics form. Interviews were scheduled at a convenient time and each patient was asked to verbally re-confirm their consent to take part at the start of their interview. Semi-structured interviews (1 hour in duration) were conducted via teleconference by trained qualitative interviewers (all of whom were members of the Adelphi Values project team); no repeat interviews were conducted. Due to the qualitative research previously conducted during the development of the ADSD and ANSD [10, 11], the concept elicitation section of the interview was relatively brief (approximately 10 minutes) and aimed to explore patients’ experience of asthma, including symptoms and health-related quality of life impacts. Open-ended questions were used to encourage spontaneous conversation and help elicit and identify the concepts most important to patients, in their own words. Patients were also asked more focused questions designed to probe on concepts that they may not have mentioned during the interview or concepts/statements that required additional clarification. This section of the interview took place prior to patients seeing the ADSD and ANSD items to ensure that they were not biased by the content of the diaries.

The cognitive debriefing section of the interview (approximately 45 minutes) used detailed questions to examine the relevance of the ADSD and ANSD concepts to patients and their understanding of the instructions, items, response options, and associated recall periods. Patients were instructed to use a ‘think-aloud’ approach when answering the questions; they were also asked to provide general feedback on the measures. In addition, patients’ perceptions of meaningful changes in item scores on the ADSD and ANSD were investigated. As noted earlier, the ADSD assessed the timeframe between a patients’ waking and going to bed (i.e., the daytime period), while the ANSD assessed the timeframe between a patients’ going to bed and waking (i.e., the nighttime period) [11]. Due to time constraints during the interviews, it should be noted that ADSD/ANSD supplementary items assessing limitations in usual activities, nighttime awakenings and medication use were not debriefed.

### Data analysis

#### Patient characteristics

Sociodemographic and clinical characteristics collected from the patient-completed screener and demographics forms were summarized using descriptive statistics.

#### Concept elicitation and cognitive debriefing interviews

Qualitative analysis of verbatim transcripts for the interviews was conducted based on a qualitative analysis plan developed a priori. Patient quotations were sorted by domain using thematic analysis methods, and codes pertinent to the main research questions were applied. An axial coding process enabled the relationship between concepts to be explored. The coding scheme was developed by the research team and was adapted as needed during the remainder of the analysis process. The verbatim transcripts were stored, coded, and analyzed using ATLAS.ti software [19].

Briefly, the first two transcripts were coded by two members of the coding team separately. The Adelphi Values project leader reviewed the analysis of the first interviews with the project researchers and any feedback was incorporated into these and future transcripts. Coding and the identification and development of themes (thematic analysis) involved a combined inductive-deductive analysis approach. A preliminary coding dictionary outlined a list of predetermined codes that were developed based on prior knowledge, evidence from the literature, study objectives and the research question (deductive analysis). However, the coding dictionary evolved iteratively throughout coding and analysis as new concepts were identified from the data (inductive analysis).

Each subsequent transcript (after the first two) was coded by individual members of the research team, but transcripts selected at random were quality checked by the project leader. Prior to coding an interview, each coder read through the transcript in its entirety, points were highlighted as a comment or code in ATLAS.ti and any discrepancies were resolved through a discussion and consensus-building process. As additional codes were added, previously coded transcripts were revisited and reviewed to identify any instances where the new code may have applied. Following the review and coding of all qualitative data, all coded data, themes and supporting quotes were extracted from ATLAS.ti into an Excel data extraction spreadsheet. Patients did not receive interview transcripts or provide feedback on these. Quotations are included in the supplementary appendices.

A conceptual saturation analysis was also conducted for asthma symptoms captured during the concept elicitation section of the interview, in order to justify sample size and also to confirm if the most important concepts had been identified. Concepts elicited during this section of the interview were compared between subgroups of patients using a stepwise approach. Patients were divided into three equal groups (in chronological order, by order in which the interviews were conducted). Concepts reported by the first set of patients (i.e., interviews 1–5) were compared with the concepts elicited by the next set of patients (i.e., interviews 6–10). The consolidated list of concepts reported from the first two sets of patients was compared with concepts reported from the third set of patients (i.e., interviews 11–15). At each step, any new concepts that emerged from the previous set of interviews were documented. A saturation grid was developed to summarize the results of the analyses. Responses elicited from every interview were compared with prior groups to support the achievement of saturation (i.e., consistency in reported concepts between groups and whether any new concepts or sub-concepts were elicited). The focus on core symptoms of asthma meant that spontaneous elicitation of additional concepts (such as those representative of comorbid conditions or clinically-unrelated to asthma) for the first time in the last set of interviews was not considered as evidence of saturation not having been met or additional interviews being required.

Perceptions of what would constitute a meaningful change in ADSD and ANSD scores (at the item level) were also explored qualitatively. Patients selected a response option for each item and were asked, based on their chosen response, what level of change would constitute a meaningful improvement or worsening. A separate total score (daily average) was calculated (ranging from 0 to 10 and derived by averaging scores across all 6 ADSD/ANSD items) and the patient was informed of their score. They were then asked to consider what level of improvement or worsening in the total score would be meaningful (i.e., clinically relevant) to them. Average total scores and perceived meaningful improvements at item level (in terms of point increase/reduction) are reported here for all patients who provided this information. Due to time constraints during the interview, not all patients were asked about meaningful levels of improvement and worsening on the ADSD and ANSD.

#### Ethics

The study was conducted in accordance with the principles outlined in the Declaration of Helsinki. Institutional review board approval for the qualitative interview was obtained from Western Copernicus Group Independent Review Board (approval code 45168598). Ordinal patient identification numbers were assigned herein to provide context for quotations while maintaining anonymity.

## Results

### Patient population

A total of 15 adults with moderate-to-severe asthma were recruited for participation in the combined concept elicitation and cognitive debriefing interviews; the sociodemographic and clinical characteristics of the study population are shown in Table 1. The majority of patients were female (*n* = 13/15) and the mean age was 47 years (range: 33–63 years). Most patients (*n* = 12/15) had been diagnosed with asthma more than 20 years prior to the study; 11/15 patients had experienced two or more severe asthma exacerbations in the 12 months prior to screening. Based on Asthma Control Test scores at screening, all patients were categorized as having either ‘poorly controlled asthma’ or ‘very poorly controlled asthma’. No barriers to ADSD or ANSD completion were reported by patients.

**Table 1:** Patient sociodemographic and clinical characteristics

	N=15
Age, years, mean (range)	47 (33–63)
Sex, n (%)	
Female	13 (87)
Male	2 (13)
Race, n (%)	
Black or African American	4 (27)
White	11 (73)
Ethnicity, n (%)	
Hispanic, Latino of Spanish origin	2 (13)
Non-Hispanic, Non-Latino or Non-Spanish origin	13 (87)
Highest education level, n (%)	
Graduate degree	3 (20)
Undergraduate degree	4 (27)
College or Associate degree	7 (47)
Middle and High School	1 (7)
Work status, n (%)	
Working full time*	9 (60)
Full-time homemaker	2 (13)
Full-time student	1 (7)
Part-time student	1 (7)
Unable to work due to asthma	1 (7)
Unable to work unrelated to asthma	1 (7)
Time since asthma diagnosis, n (%)	
7–10 years	1 (7)
11–20 years	2 (13)
21–40 years	9 (60)
>41 years	3 (20)
ACT score, mean (range)	14 (9–20)
ACT classification, n (%)	
Very poorly controlled (0–15)	9 (60)
Poorly controlled (16–20)	6 (40)
Well-controlled (21–25)	0 (0)
GINA step, n (%)	
4	11 (73)
5	4 (27)
No. of asthma exacerbations^†^ in the past 12 months, n (%)	
1 exacerbation	4 (27)
2 exacerbations	11 (73)
ICS dosage classification, n (%)	
Medium	11 (73)
High	4 (27)
Diagnosis of allergic rhinitis, n (%)	7 (47)

*In a paid job or as a volunteer. ^†^Defined by the requirement for addition of or increase in the dose of oral corticosteroid treatment, and/or injection of corticosteroid, and/or visits to the emergency room and/or hospitalization due to asthma symptoms. ACT, Asthma Control Test; GINA, Global Initiative for Asthma; ICS, inhaled corticosteroid.

### Content validity

#### Concept elicitation interview findings: asthma symptoms and impacts

**Fig. 2:**
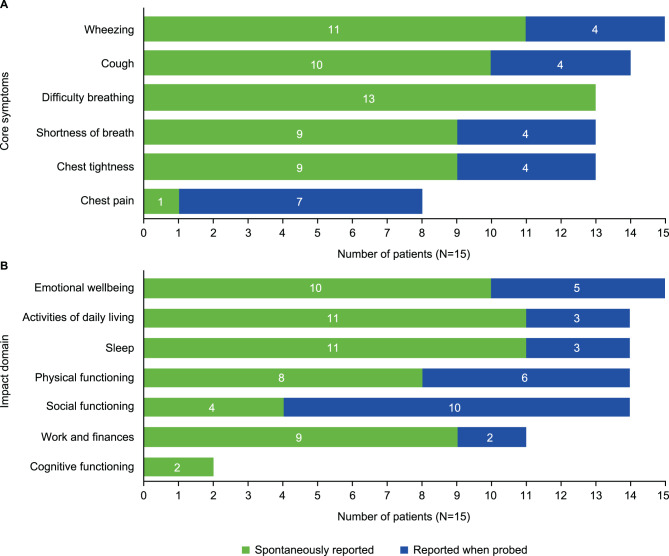
(**A**) Symptoms and (**B**) impacts reported during concept elicitation section of interview

Symptoms assessed by the ADSD and ANSD were those most frequently reported by patients during open-ended questioning. Core asthma symptoms of wheezing (*n* = 15/15), cough (*n* = 14/15), difficulty breathing (*n* = 13/15), shortness of breath (*n* = 13/15), and chest tightness (*n* = 13/15) were reported by all or almost all patients, with the majority of patients verbalizing these symptoms spontaneously (Fig. 2A). Additionally, chest pain was reported by more than half of patients (*n* = 8/15). The most bothersome symptoms reported were shortness of breath (*n* = 5/12), wheezing (*n* = 4/12), difficulty breathing (*n* = 3/12), and chest tightness (*n* = 3/12); some patients reported multiple symptoms as being most bothersome. Analysis indicated that conceptual saturation was achieved; all key symptoms of asthma were identified by the sample of patients interviewed (see Supplementary Methods for details).

All patients (*n* = 15/15) discussed the impact that asthma symptoms had on various aspects of their daily lives. The reported impacts could be broadly categorized into seven domains (Fig. 2B). The most frequently reported impact domains were emotional well-being (*n* = 15), and activities of daily living, sleep, physical functioning, and social functioning (*n* = 14 per domain).

#### Cognitive debriefing interview findings: relevance and understanding of the ADSD and ANSD items and instructions

**Fig. 3:**
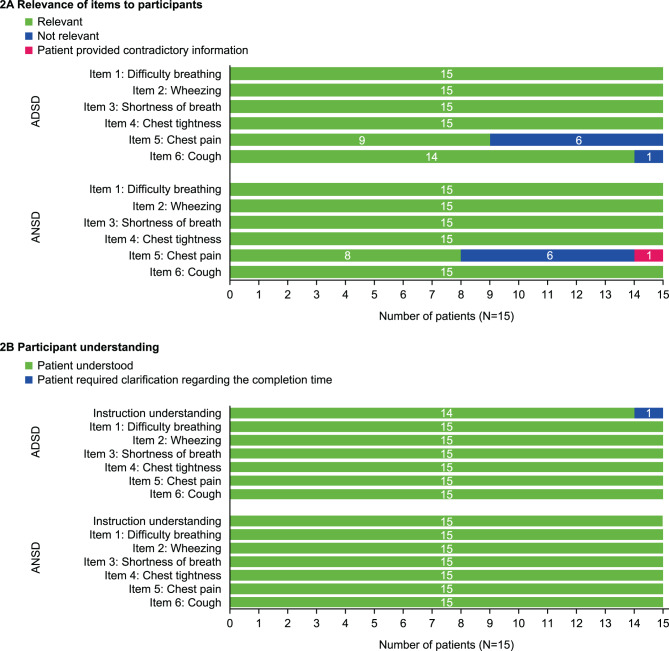
Patient assessment of (**A**) item relevance and (**B**) understanding of ADSD and ANSD. ADSD, Asthma Daytime Symptom Diary; ANSD, Asthma Nighttime Symptom Diary

All patients (*n* = 15/15) indicated that they experienced difficulty breathing, wheezing, shortness of breath, and chest tightness due to their asthma during the day and night (Fig. 3A); cough was experienced by all patients at night (*n* = 15/15), with most also reporting experiencing cough during the day (*n* = 14/15). Most patients had experienced chest pain during the day (*n* = 9/15) and night (*n* = 8/15; Fig. 3A). All patients (*n* = 15/15) demonstrated a good understanding of the ADSD and ANSD items and instructions; clarification regarding the proposed timing of completion was required by one patient (Fig. 3B). Detailed patient feedback on their understanding and the relevance of ADSD and ANSD items is provided in Table E1 and Table E2.

#### Cognitive debriefing interview findings: appropriateness of the recall period and meaningful change

**Fig. 4:**
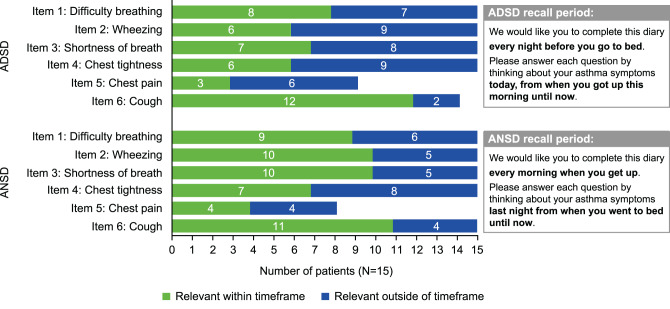
Appropriateness of recall period of ADSD and ANSD. ADSD, Asthma Daytime Symptom Diary; ANSD, Asthma Nighttime Symptom Diary

The recall period of the ADSD and ANSD was deemed appropriate as most patients had either experienced symptoms within the specified period or noted that they ‘typically’ experienced symptoms during the day/night (Fig. 4; also described in Tables E1 and E2).

**Fig. 5:**
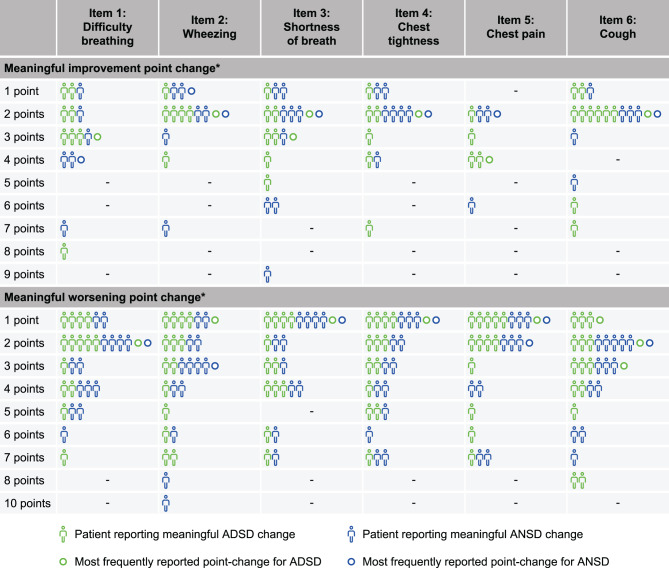
Item-level meaningful change in score analysis for ADSD and ANSD. The number of patients who provided meaningful change data varied per item. *Response options on the ADSD and ANSD range from 0 (none) to 10 (as bad as you can imagine). ADSD, Asthma Daytime Symptom Diary; ANSD, Asthma Nighttime Symptom Diary. Each human symbol represents one patient’s feedback and circles indicate the point-changes indicated by the highest number of patients for each item. Human symbols and circles are color-coded as light blue for ADSD and dark blue for ANSD

Meaningful change in ADSD and ANSD total scores was explored among a subset of interviewed patients. The average point improvement considered meaningful on the ADSD total score (daily average across all 6 ADSD item scores) was 1.7 (based on data from 8/15 patients; range 0.5–3.5). Reductions of 0.5–1.0-points (*n* = 3/8) and 1.1–2.0-points (*n* = 3/8) in ADSD total score were most frequently reported by patients to be meaningful. The average point worsening considered meaningful was 2.8 (based on data from 11/15 patients; range 1.0–5.8), with patients most commonly reporting that a 1.1–2.0-point (*n* = 3/11) or 2.1–3.0-point (*n* = 3/11) increase in ADSD total score would be meaningful. The average point improvement considered meaningful in ANSD total score (daily average across all 6 ANSD scores) was 2.4 (based on data from 10/15 patients; range 0.2–5.0). Reductions of 0.2–1.0-points, 1.1–2.0-points, or 4.1–5.0-points were each considered meaningful by 3 patients. The average level of point worsening that would be considered meaningful was 2.3 (based on data from 11/15 patients; range 0.5–6.7). Patients most commonly reported that a 0.5–1.0-point (*n* = 4/11) or a 2.1–3.0-point (*n* = 3/12) increase in ANSD total score would be meaningful to them. Item-level meaningful change in scores for ADSD and ANSD are shown in Fig. 5. When considering individual items, a 2-point reduction would be the smallest change considered to be a meaningful improvement (ADSD and ANSD: wheezing, shortness of breath [2/3 points for ADSD], chest tightness and cough; ANSD only: chest pain). Slightly larger reductions were associated with meaningful improvement for difficulty breathing (ADSD: 3 points; ANSD: 4 points) and chest pain (ADSD: 4 points). A 1-point increase was the smallest change representing meaningful worsening (ADSD and ANSD: shortness of breath, chest tightness and chest pain; ADSD only: wheezing (1-point) and cough [1–3 points for cough]). A 2-point increase was linked to meaningful worsening for difficulty breathing and cough for both the ADSD and ANSD, and to chest pain for the ANSD only; a 3-point increase was perceived as meaningful worsening for wheezing with the ANSD.

#### Cognitive debriefing interview findings: general feedback on the ADSD and ANSD

All patients (*n* = 15/15) indicated that the length of the diaries was appropriate. All nine patients asked (*n* = 9/9) reported that it would be feasible to complete the diaries daily as part of a 3- or 6-month clinical study, due the perceived short completion time.

## Discussion

The ADSD and ANSD were developed through prior qualitative research in adults and older adolescents with mild-to-severe asthma [10, 11]. The current study provides empirical qualitative evidence supporting the content validity of the ADSD and ANSD specifically among patients with moderate-to-severe asthma. Qualitative data exploring meaningful change for ADSD and ANSD total scores also supports quantitative evidence to establish measurement properties of the ADSD and ANSD in this target population.

Consistent with initial research during measure development, the most frequently reported symptoms of moderate-to-severe asthma in the concept elicitation section of the interviews were those represented by the items in the measures (i.e., difficulty breathing, wheezing, shortness of breath, chest tightness, chest pain, and cough) [10, 11]. Saturation analyses indicated that the six core symptoms of asthma were identified in the interview sample and confirmed the relevance of symptoms across patients with moderate-to-severe asthma. Patients also reported a range of impacts due to asthma (particularly on emotional well-being, activities of daily living, sleep, physical functioning, and social functioning) that were consistent with those experienced by the broad asthma population included in prior ADSD and ANSD research [10, 11]. Patients reported differences in the experience and impact of asthma symptoms in the daytime and nighttime (including cough being more frequently reported at night), highlighting the value of independent assessment of the severity of daytime and nighttime asthma symptoms by the ADSD and ANSD, respectively; notably, the ANSD is the only asthma patient-reported score which assesses nighttime symptoms independently [8]. Although the ADSD and ANSD scores are generally used together, the fact that they assess distinct time periods does allow the possibility for them to be used separately (e.g., in patients who predominantly experience nighttime symptoms).

In line with previous findings, patients in this study demonstrated a good understanding of the ADSD and ANSD, with minimal exceptions. There appeared to be no clear differences in how individuals with moderate-to-severe asthma interpret or understand the ADSD and ANSD compared with the broader asthma population [11]. Patients were able to discriminate between the items and response options within each diary and all symptoms assessed were deemed relevant to their experience of asthma during the daytime or nighttime. As expected based on the concept elicitation findings and earlier results [10], chest pain was less relevant to some patients; however, it was reported by more than half of the sample, suggesting its continued relevance. The respective recall periods of the ADSD and ANSD were also deemed appropriate to the study sample as most patients either confirmed they had experienced the symptom within the specified period or within this timeframe on a ‘typical day/night’. It was noteworthy that for the ADSD relative to the ANSD, a greater proportion of patients considered the items relevant outside of the timeframe specified; this likely reflects variation in asthma symptoms from day to day. The use of daily diaries and the calculation of an average weekly score would be better suited to help capture daily fluctuation in symptoms (including any symptom-free days) compared with a 7-day recall period, as well as the measuring the severity of symptoms that are experienced on a less frequent basis while minimizing recall bias [5].

As noted earlier, quantitative evaluations of the ADSD and ANSD measurement properties have recently been performed in a real-world observational study and a randomized controlled trial of patients with asthma, including analyses of cross-sectional and longitudinal validity and estimates of meaningful change thresholds [13]. To support quantitative findings, we qualitatively explored meaningful change in ADSD and ANSD scores in the Cognitive debriefing interview section to provide insights into improvement and worsening of scores that the patients with moderate-to-severe asthma deemed to be relevant to them. It was generally agreed that a 1- to 2-point change in score was relevant. These data are important because differences in the frequency and severity of symptoms in moderate-to-severe patients may have implications for definitions of meaningful within-patient change compared with a broad or milder asthma patient population.

When interpreting these results there are a few potential limitations to consider. Firstly, the fact that the majority of patients were White, female and of non-Hispanic, non-Latino or non-Spanish origin may limit the representativeness of these results. Secondly, time constraints during the interview meant that not all patients were asked about meaningful change for all ADSD and ANSD items. Also, meaningful change was explored at a single timepoint and ADSD/ANSD summary scores were evaluated based on responses to a single day; however, it is anticipated that weekly mean scores will be used for primary or secondary efficacy endpoints in confirmatory studies. The majority of the patients also described their symptoms as ‘better than usual’ on the day of their interview, with relatively mild symptoms that scored at the lower end of the response scales. Point estimate changes, therefore, may have been different had patients been experiencing more severe symptoms on the day of the interview. Also, it should be noted that no conclusions could be drawn on interpretation of meaningful change by asthma severity (i.e., moderate versus severe); the sample size was not sufficient to perform sub-group analyses. Nonetheless, the estimates reported here are considered valuable for supporting traditional anchor- and distribution-based quantitative estimates of meaningful change as explored based on longitudinal ADSD and ANSD collected as part of real-world observational studies and randomized controlled trials in patients with moderate to severe asthma. Estimates of meaningful change are necessary to accurately assess the extent of treatment benefit in different therapy areas, and also provide a useful basis for the design and interpretation of results from clinical trials; therefore, studies exploring meaningful change scores are essential for optimization of clinical research [20].

## Conclusions

This qualitative, semi-structured interview study supports the content validity of the ADSD and ANSD in adults and older adolescents with moderate-to-severe asthma, providing important qualitative evidence to inform the estimation of minimal clinically important difference. The ADSD and ANSD are valuable additions to existing PROs in asthma as measures of asthma symptom severity. When considered alongside extensive prior research and quantitative analyses, the findings will facilitate the use of the diaries in clinical trials assessing intervention efficacy and provide useful insights into the extent of asthma symptoms and aid clinical management in real-world clinical practice.

## Electronic supplementary material

Below is the link to the electronic supplementary material.

Supplementary Material 1

## Data Availability

GSK makes available anonymized individual patient data and associated documents from clinical studies upon approval of proposals submitted to: https://www.gsk-studyregister.com/en/.

## Abbreviations

ACT: Asthma Control Test
ADSD: Asthma Daytime Symptom Diary
ANSD: Asthma Nighttime Symptom Diary
CD: Cognitive debriefing
CE: Concept elicitation
FDA US: Food and Drug Administration
GINA: Global Initiative for Asthma
ICS: Inhaled corticosteroid
PRO: Patient-reported outcome
US: United States
