# Supplementary material for: Asthma daytime and nighttime symptom diaries: content validity and qualitative exploration of meaningful change in patients with moderate-to-severe asthma
Source: J Patient Rep Outcomes. 2026 May 28;10:90. doi: 10.1186/s41687-026-01079-0 (PMC13219637; doi:10.1186/s41687-026-01079-0)
Supplement: Supplementary file 1 — Supplementary Material 1 [file 41687_2026_1079_MOESM1_ESM.docx]

Supplementary content

*Table E1. Patient feedback on understanding and relevance of ADSD items*

| Item | Understanding | Relevance |  |  |
| --- | --- | --- | --- | --- |
| 1 | Please rate your **difficulty breathing** at its worst since you **got up this morning** (0=None, 10=As bad as you can imagine) | |  |  |
|  | 14/15 patients demonstrated an understanding of the item as intended and selected an appropriate response, e.g.,“I would probably say…a four would be a better gauge of it because it did cause me to catch my breath when I was outside, um, because it is colder here today than it has been”**Interviewer: “Okay. And can you explain why you chose a zero for that one?”** “Uh, not had any difficulty breathing since I work up this morning”1 patient did not provide an explanation for their response but did show understanding of the response options and directionality as on a typical day they would have selected a three:“I would say six…Three regularly” | 15/15 patients reported that difficulty breathing was relevant to their experience of asthma during the day8/15 had experienced difficulty since they got up, e.g., “I am having a little more difficulty breathing it seems in like in the last, um, couple of hours. So that’s why I would pick a six as opposed to something lower”7/15 reported that they do experience difficulty breathing but not on the day of the interview, although they did have this on a typical day, e.g., “For me, I’d have to say zero. I’ve been really good this morning”. **Interviewer: “And how would you rate your difficulty breathing at its worst on a typical day?”** “On a typical day, I would say somewhere between a zero and a two” |  |  |
| 2 | Please rate your **wheezing** at its worst since you **got up this morning** (0=None, 10=As bad as you can imagine) | |  |  |
|  | 14/15 patients demonstrated an understanding of the item as intended and selected an appropriate response, e.g.,“…that’s maybe a two. I haven’t had much this morning”1 patient did not contextualize their response, i.e.,“It’s been zero” | 15/15 patients reported that wheezing was relevant to their experience of asthma during the day6/15 patients indicated that they experienced wheezing within the timeframe specified in the item, e.g., “I would say it’s probably a four…I think I’ve only wheezed a couple of times since I, I work up”9/15 patients reported that they do experience wheezing but not on the day of the interview. 4/9 had wheezing on a typical day, e.g., “I would have to say zero because I, I don’t have any wheezing this morning”. **Interviewer: “Then how would you rate your wheezing at its worst on a typical day?”** “Probably somewhere around a four or a five” |  |  |
| 3 | Please rate your **shortness of breath** at its worst since you **got up this morning** (0=None, 10=As bad as you can imagine) | |  |  |
|  | 13/15 patients demonstrated an understanding of the item as intended and selected an appropriate response, e.g.,“…please rate your shortness of breath, I would put it at a, a zero, I did not feel short of breath”2 patients did not explain why they selected their response, i.e.,“Um, seven”“That’s a zero as well. Yeah” | 15/15 patients indicated that shortness of breath was experienced during the day6/15 patients indicated that they experienced wheezing within the timeframe specified in the item, e.g., “I’ve been having struggling with breath since I got up. I’ve been having trouble.  My breathing is hard”8/15 patients reported that they do experience shortness of breath but not on the day of the interview. 3/8 had wheezing on a typical day, e.g., “It’ll be a zero. Um, but on a, an average day, it’ll be between two and three, the shortness of breath. All depends on that specific day” |  |  |
| 4 | Please rate your **chest tightness** at its worst since you **got up this morning** (0=None, 10=As bad as you can imagine) | |  |  |
|  | 14/15 patients demonstrated an understanding of the item as intended and selected an appropriate response, e.g.,“Probably a two…Yeah. It’s just a little bit of, a little bit of constriction when I got up this morning. Um, and then after I did my nebulizer, it, it loosened”1 patient did not explain their response, i.e.,“Six”. **Interviewer: “Six. Okay. Any what would you rate [chest tightness]…at its worst on a typical day?”** “Eight” | 15/15 patients indicated that chest tightness was experienced during  the day6/15 patients indicated that they experienced wheezing within the timeframe specified in the item, e.g., “Uh, maybe a two, three. Um, it hasn’t been that bad. So I did use my inhaler this morning…”9/15 patients reported that they do experience chest tightness but not on the day of the interview. 1 patient indicated that they would rate their chest tightness 1–2 on a typical day but did not report it during the interview. 2/9 patients reported that they do have this symptom on a typical day, e.g., “So again, this morning, zero. But on a typical day, um, it’ll be like a three or four” |  |  |
| 5 | | Please rate your **chest pain** at its worst since you **got up this morning** (0=None, 10=As bad as you can imagine) | |  |
|  | | 15/15 patients demonstrated an understanding of the item as intended and selected an appropriate response, e.g.,“I would say a four. I don’t really have that much chest pain as of right now” | 9/15 patients indicated that chest pain was experienced during the day3/9 patients indicated that they experienced chest pain since they got up, e.g., “Chest tightness and chest pain are very similar, but I mean I would say if they are I’m going to go with six”6/9 patients reported that they do experience chest tightness but not on the day of the interview; 1 of these indicated that they would rate their chest pain 4–5 on a typical day. 3/6 patients reported that they do have this symptom on a typical day, e.g., “This morning, no chest pain. Um, on a typical asthma day, chest pain would probably run at again a six or a seven”6/15 patients indicated that chest pain was not relevant to their experience of asthma as they never had this symptom, e.g.,“This gets a zero, because that’s never been a symptom” |  |
| 6 | | | Please rate your **cough** at its worst since you **got up this morning** (0=None, 10=As bad as you can imagine) | |
|  | | | 15/15 patients demonstrated an understanding of the item as intended and selected an appropriate response, e.g.,“Probably a three…it woke me up this morning and it’s just been kind of…there today…Cough is one of my bigger symptoms”“It’s been about a, a three, cause I’ve been coughing quite a bit this morning” | 14/15 patients indicated that chest pain was experienced during the day12/14 patients indicated that they experienced chest pain since they got up, e.g., “I would say my cough is probably at about a five this morning, um, because I did wake up and have some coughing and then I had the coughing when I went outside as well”2/14 patients reported that they do experience cough during the day but not on the day of the interview, e.g., **Interviewer: “How often do you…have a cough during the day because of your [asthma]?”** “Maybe, every few weeks or so”1 patient indicated that cough was not relevant to their experience of asthma during the day as they only experienced the symptom at night, i.e., “That’s a zero. And like I said, because for me, I don’t normally have a cough except at night” |

ADSD, Asthma Daytime Symptom Diary.

## Table E2. Patient feedback on understanding and relevance of ANSD items

| Item | Understanding | Relevance |
| --- | --- | --- |
| 1 | Please rate your **difficulty breathing** at its worst since you **went to bed last night** (0=None, 10=As bad as you can imagine) | |
|  | 14/15 patients demonstrated an understanding of the item as intended and selected an appropriate response, e.g.,   - *“It wasn’t as bad last night thankfully than it had been previous nights this week. I woke up a few times last night, so I didn’t think it was as bad as some nights where I’m up five to ten times. It was probably just three times last night, so that’s why I picked a seven”*   One patient did not provide an explanation for their response:   - *“Please rate your difficulty breathing, breathing at its worst since you went to bed last night…Six.”* **Interviewer:** *“****How much would this score have to worsen for you to consider it an important worsening in your difficulty in breathing during the night?****” “Eight”* | 15/15 patients reported that difficulty breathing was relevant to their experience of asthma during the night   - 9/15 reported that they experienced difficulty breathing since they went to bed, e.g., *“Five…’Cause I was up twice last night”* - 6/15 patients reported that they do experience difficulty breathing on a typical night but had not experienced the symptom during the night prior to the interview. However, 3/6 reported that they have difficulty breathing on a typical night   - *“For me right now that would be a zero for last night. Um, I didn’t have any symptoms last night…”* ***Interviewer:*** *“****Okay. And is difficulty breathing, is that something that you ever experience at nighttime?****” “Yeah. Oh yeah. For sure. Yeah. “* ***Interviewer:*** *“****How would you rate your difficulty breathing at its worst on a typical night?****” “Uh, eight.”* |
| 2 | Please rate your **wheezing** at its worst since you **went to bed last night** (0=None, 10=As bad as you can imagine) | |
|  | 13/15 patients demonstrated an understanding of the item and selected an appropriate response, e.g.,   - *“Again, I would say it was a two because there was wheezing present, but it wasn’t enough to, um, cause me to have an asthma attack”* - 2 patients did not provide an explanation for the response they selected, e.g.,   - *“Right now, well last night again, it was at a two”*   - *“Um, seven”* ***Interviewer:*** *“****And how much would it have to improve for you to consider it an important improvement on your wheezing during the night?****” “Um, nine”* ***Interviewer:*** *“****So, just to remind you that the scale-so zero would be none and ten would be as bad as you can imagine****” “Then we’d have to say four”* | 15/15 patients indicated that wheezing was relevant to their experience of asthma during the night   - 10/15 patients reported that they experienced wheezing in the timeframe specified, e.g., *“I would say it was a two because there was a wheezing present, but it wasn’t enough to, um, cause me to have an asthma attack…it’s something that’s there, um, just about every night, but I wouldn’t say that it’s something that’s horribly terrible every single night”* - 5/15 patients reported that they did experience wheezing at night but had not during the night before the interview. However, 1 of these patients reported that wheezing is experienced on a typical night, e.g., *“I didn’t have any wheezing last night, so I would say a none, 0. A 0, yeah…[I experience it] maybe once or twice, um, in a week”* |
| 3 | Please rate your **shortness of breath** at its worst since you **went to bed last night** (0=None, 10=As bad as you can imagine) | |
|  | 13/15 patients demonstrated an understanding of the item and selected an appropriate response, e.g.,   - *“I would say probably about a three, um, because I did have some shortness of breath, um, but it wasn’t a full asthma attack that I encountered, just the fact that there was some, um, feeling of, of not having full capacity of breathing”* - 2 patients did not provide an explanation for the response selected but showed understanding of the response options and directionality when discussing meaningful change, e.g., *“I’m going to say eight.”* ***Interviewer: “So how much would it have to improve for you to consider an important improvement?”*** *“A zero to a one.”* ***Interviewer: “How would you rate your shortness of breath at it, at its worst on a typical night?”*** *“Five”* | 15/15 patients reported that shortness of breath was relevant to their experience of asthma during the night   - 10/15 indicated that they experienced shortness of breath during the night before the interview, e.g.,  *“I would say probably about a five…So my shortness of breath isn’t as bad as I would say a normal night where it’d be more like a seven”* - 5/15 patients reported that they do experience shortness of breath at night but had not experienced the symptom during the night prior to the interview. However, 2/5 reported that shortness of breath is experienced on a typical night, e.g., *“I didn’t have much shortness of breath, I would say, um, 0. Yeah.”*  ***Interviewer:*** *“****And is that something you ever experience at night?****” “Some nights, maybe once or twice in a week’s period”* |
| 4 | Please rate your **chest tightness** at its worst since you **went to bed last night** (0=None, 10=As bad as you can imagine) | |
|  | 14/15 patients demonstrated an understanding of the item and selected an appropriate response, e.g.,   - *“That was two. It was really minimal last night, the chest tightness. Um, yeah. It just didn’t, didn’t wake me up. When I woke up, it wasn’t noticeable”*   One patient did not explain why they selected the response but understood the response options and directionality when discussing meaningful change, i.e., *“Uh, five”* ***Interviewer: “…how much would it have to improve for you to consider it an important improvement?”*** *“Um, three.”* ***Interviewer: “And how much would it have to worsen for you to consider it an important worsening?”*** *“Seven”* | 15/15 patients reported that chest tightness was relevant to their experience of asthma during the night   - 7/15 patients reported that they had chest tightness since they went to bed, e.g., *“That was probably at a three…Um, cause I mean, it did wake me up”* - 8/15 patients reported that they did experience chest tightness at night but had not experienced the symptom during the night before the interview. However, 3/8 of these patients reported that chest tightness is experienced on a typical night. 1 patient expressed that chest tightness was not a common symptom for them at night during the CD section of the interview but indicated that the symptom had never been experienced due to asthma during the CE section of the interview, i.e., *“Last night, I didn’t have that. So, it’s a zero, happily. The chest tightness goes along with all of the, the three earlier thingies, when they’re, when they’re becoming a problem or, a serious issue, you know, it’s, all of those things happen”* |
| 5 | Please rate your **chest pain** at its worst since you **went to bed last night** (0=None, 10=As bad as you can imagine) | |
|  | 14/15 patients demonstrated an understanding of the item and selected an appropriate response, e.g.,   - *”I’d have to say it was a two, because I didn’t have much”*   1 patient did not explain their response but understood response options and directionality when discussing meaningful change, i.e.,   - *“Um, four”* ***Interviewer: “And, um, how much would it have to improve for your to consider it an important improvement?”*** *“Two”* ***Interviewer: “And how much would it have to worsen for you to, to consider it an important worsening?”*** *“Six”* | 8/15 patients reported that chest pain was relevant to their experience of asthma during the night   - 4/8 patients reported that they had chest pain since they went to bed, e.g., *“I’d have to say a two, because I didn’t have much”* - 4/8 patients reported that they had chest pain at night but not during the night before the interview;  1 of these patients said they had chest pain on a typical night, i.e., *“A zero. I didn’t experience any of that.* ***Interviewer: “How often do you experience chest pain at night?”*** *“Uh, maybe once a month maybe…”*   6/15 patients reported that chest pain is not relevant to their experience of asthma as they have never had this, e.g.,   - ***Interviewer: “And I know, um, you said that’s not a typical symptom you suffer from. Is it something you’ve never experienced as part of asthma?”*** *“I don’t think so. I don’t think I’ve ever had pain. I’ve – you know, the feeling of tightness, yes. But I mean I don’t think ever pain, no. Not at all”*   1 patient provided contradictory information, i.e.,  *“I didn’t have any chest pain last night”.* ***Interviewer:  “Do you ever experience chest pain because of your asthma?”*** *“I have once or twice”* |
| 6 | Please rate your **cough** at its worst since you **went to bed last night** (0=None, 10=As bad as you can imagine) | |
|  | 14/15 patients demonstrated an understanding of the item and selected an appropriate response, e.g.,   - *”I’m going to say 10…It’s very uncomfortable…When you’re having an actual coughing fit, um, which I had several last night, you can’t get a breath”*   1 patient did not explain their response but understood response options and directionality when discussing meaningful change, i.e., *“Um, three”* ***Interviewer: “And, um, how much will that have to improve for your to consider it an important improvement?”*** *“One”* ***Interviewer: “How much would it have to worsen for you to, to consider it an important worsening?”*** *“Uh, five”* | 15/15 patients reported that cough was relevant to their experience of asthma during the night   - 11/15 patients reported that they had cough during the night prior to the interview, e.g., *“Probably a four…Um, it woke me up a couple times”* - 4/15 patients reported that they experienced cough at night but not during the night before the interview; 1 of these patients said they had cough on a typical night, i.e., *“I didn’t have any coughing last night”.* ***Interviewer: “Is that something you ever experienced?”*** *“Yes, I have. Yes I have in the past”*   6/15 patients reported that chest pain is not relevant to their experience of asthma as they have never had this, e.g.,   - ***Interviewer: “And I know, um, you said that’s not a typical symptom you suffer from. Is it something you’ve never experienced as part of asthma?”*** *“I don’t think so. I don’t think I’ve ever had pain. I’ve – you know, the feeling of tightness, yes. But I mean I don’t think ever pain, no. Not at all”*   1 patient provided contradictory information, i.e., *“I didn’t have any chest pain last night”.* ***Interviewer: “Do you ever experience chest pain because of your asthma?”*** *“I have once or twice”* |

ANSD, Asthma Nighttime Symptom Diary.
